# Duckweed Is a Promising Feedstock of Biofuels: Advantages and Approaches

**DOI:** 10.3390/ijms232315231

**Published:** 2022-12-03

**Authors:** Gui-Li Yang

**Affiliations:** 1 Key Laboratory of Plant Resource Conservation and Germplasm Innovation in Mountainous Region (Ministry of Education), Collaborative Innovation Center for Mountain Ecology & Agro-Bioengineering (CICMEAB), College of Life Sciences/Institute of Agro-Bioengineering, Guizhou University, Guiyang 550025, China; glyang3@gzu.edu.cn; 2 Institute of Geochemistry, Chinese Academy of Sciences, Guiyang 550081, China

**Keywords:** biomass, duckweed, energy plants, starch, pathway

## Abstract

With the growing scarcity of traditional sources of energy and the accompanying acute environmental challenges, biofuels based on biomass are favored as the most promising alternative. As one of the core raw materials for biomass energy, research on its production methods and synthesis mechanisms is emerging. In recent years, duckweed has been used as a high-quality new biomass feedstock for its advantages, including fast biomass accumulation, high starch content, high biomass conversion efficiency, and sewage remediation. This study provides a systematic review of the growth characteristics, starch metabolism pathways, and methods to improve starch accumulation in the new energy plant, duckweed. The study also presents a prospect that might be used as a reference for the development of duckweed as a new energy-providing plant.

## 1. Introduction

Due to the rise in energy demand, fossil energy is becoming scarce, along with an increase in eco-environmental problems. To solve this problem, researchers have started investigating the development and utilization of renewable or sustainable clean energy [1]. Biomass energy refers to the form of energy in which solar energy is converted to chemical energy and stored as biomass. This occurs directly or indirectly during photosynthesis in green plants. Biomass energy is the fourth largest energy source after coal, oil, and natural gas [2]. The carbon in biomass energy comes from CO_2_ in the atmosphere, and thus, the production and consumption of biomass energy do not increase the total carbon in the atmosphere. Hence, it is clean and renewable energy and the only carbon resource that can replace fossil energy [3]. Therefore, biomass energy is the best resource with which to deal with global climate change, energy shortage, and environmental pollution. Many countries and regions have adopted legislation to encourage or even force the promotion of biomass energy [4].

In the production of biomass energy, the cost of raw materials accounts for 70% to 80% of the total production cost, and currently, food crops such as corn, wheat, and potatoes serve as the raw materials [5]. The energy production technology based on corn and other starch resources is the most developed and has the simplest production process [6,7]. However, the large-scale promotion of food crops, which are the raw materials, can trigger the dilemma of “competing with people for food and agricultural land”, which poses a threat to global food security [8]. Thus, the industrialization of energy using a technology-oriented route might encounter problems related to the scarcity of raw materials. The biomass energy industry must shift from grain to non-grain energy plants as the main raw material [9,10]. Thus, there is an urgent need to determine non-grain energy plant resources that are suitable for new energy industrialization and large-scale development to solve the scarcity of raw materials.

*Lemnaceae*, commonly known as duckweed, has 36 species in five genera [11] and is distributed worldwide. The plants have characteristics such as high environmental adaptability, high growth rate, and high dry matter accumulation [12,13]. As an aquatic plant, duckweed grows without occupying land and can accumulate large amounts of starch, which is a raw material for producing biofuel, through phototrophic autotrophy. The average annual growth rate of duckweed can reach 12.4 g m^−2^ d^−1^ (DW), and 1 hm^2^ of duckweed can accumulate approximately 28 t of starch per year [14]. Duckweed can be converted to bioethanol with a conversion efficiency of 94.7% of the starch in duckweed [15] and a theoretical yield of ethanol about eight times that of corn [16]. Additionally, duckweed can remove pollutants from wastewater through enrichment and can combine water pollution control with bioenergy production [17,18,19]. Therefore, duckweed is a promising strategic new non-food biomaterial that might solve the feedstock problem faced during the development of the biofuel industry [20].

Several studies have expounded the research status and application value of duckweed in the fields of environmental detection, environmental restoration, and energy production [15,21,22]. However, studies are missing on the methods and principles of improving starch as an important component of biomass raw materials. Therefore, in this study, we systematically reviewed the growth characteristics, starch metabolism pathways, and methods for improving the starch accumulation of the new energy plant duckweed. The study might provide a theoretical reference for developing duckweed as a biofuel.

## 2. Biological Properties of Duckweed

Duckweed is an aquatic plant that grows rapidly year-round in temperate and tropical regions. Some species spend only 1 to 2 days for one generation in optimal conditions [12]. The structure of duckweed is simple, consisting of fronds and roots, which are green and sometimes purple on the abaxial surface. Some genera, such as *Wolffia* and *Wolffiella* [23], only have fronds, and *Wolffia* is the smallest monocotyledonous flowering plant in the world [24]. Duckweed reproduces both asexually and sexually. Under suitable conditions, duckweed reproduces asexually by budding of new shoots from the mother fronds in an exponential growth pattern. In adverse conditions, duckweed forms a dormant body (turion) that detaches from the mother and sinks into the bottom of water. It resurfaces to form a new plant when conditions are suitable [25]. Duckweed also forms seeds by flowering under environmental stress (such as nitrogen starvation) or hormonal induction (such as salicylic acid) [26,27]. Duckweed can absorb large amounts of nitrogen and phosphorus, and thus, it has a purifying effect on sewage [28]. It can also enrich toxic substances such as heavy metals in water [22]. Additionally, duckweed contains proteins, starch, and flavonoids that can be used in feed processing, new energy development, and pharmaceutical manufacturing [29,30,31]. Therefore, duckweed is a plant that can be used for environmental remediation and has gained widespread attention because of its rapid growth rate, high content of proteins and starch, and high adsorption and transfer capacity of nitrogen, phosphorus, organic matter, and heavy metals (Table 1).

### 2.1. Advantages of Duckweed as an Energy Source

**Figure 1:**
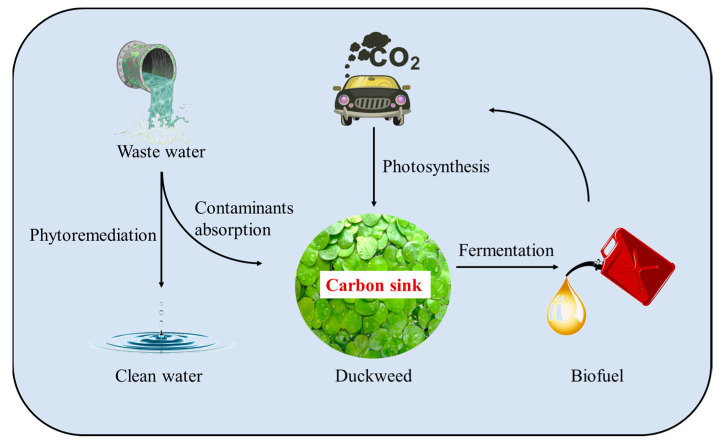
The new energy plant duckweed.

Duckweed is a starchy plant showing notable advantages, such as fast growth, low environmental requirements, no competition with people for food and land, high starch content, and low cellulose content. These properties make duckweed a kind of material for “energy gathering and environment clearing” (Figure 1).

### 2.2. High Growth Rate and Low Environmental Requirements

Duckweed is one of the fastest-growing plants, whose reproduction rate is almost close to exponential growth in low plant density, about 64 times higher than the growth rate of corn [12]. Maximum linear growth rates were observed at high plant density [37]. Duckweed has high photosynthetic efficiency and higher biomass than other plants, doubling its biomass in 16–24 h under suitable conditions. Duckweed is found in diverse ecosystems from alkaline water lakes, eutrophic water, and industrial wastewaters [38]. It has strong tolerance and growth characteristics of weak light, and it is easy to harvest when floating on the water surface, which lays the foundation for large-scale three-dimensional multi-layer culture with low cost and low energy consumption.

### 2.3. No Competition with Food Crops for Land

Duckweed is a non-food crop in most countries, unlike the large-scale production of corn, wheat, and potatoes, which may cause a food crisis. It does not compete with food crops for land [8]. Duckweed is a floating aquatic plant that grows autotrophically without occupying land or competing with land. In agricultural production, duckweed can coexist with rice, as it creates no competition with food crops for land. It also prevents nitrogen loss and improves farmland nitrogen utilization [39,40]. During sewage treatment, it can grow rapidly in water bodies, such as urban sewage and aquaculture wastewater, and purify sewage while producing non-grain starches as raw materials [41,42]. Additionally, for industrial production, high-efficiency starch production can be further achieved by establishing plant factories.

### 2.4. High Starch Content and Low Cellulose Content

The starch content of duckweed is highly variable and closely related to the duckweed variety and culture conditions, ranging from 3% to 75% of its dry weight (DW) [43]. Some varieties of duckweed can have up to six times the starch yield of maize under specific culture conditions. After artificial selection, its starch content can also reach 45% (DW) in large-scale production [14]. Moreover, duckweed has a low cellulose content, and low plant advanced structures, which makes it easy to be used for efficient fermentation. Xu et al. [15] fermented *Spirodela polyrhiza* (31% (DW) starch content) using a continuously stirred fermenter reactor, and the starch conversion efficiency was 94.7% throughout the process. This indicated that non-food duckweed starch can be converted to fuel ethanol efficiently and cost-effectively using the existing production process. Therefore, duckweed is a competitive starch source for the production of fuel ethanol and has broad development and utilization prospect.

## 3. Metabolism Pathway of Duckweed Starch

**Figure 2:**
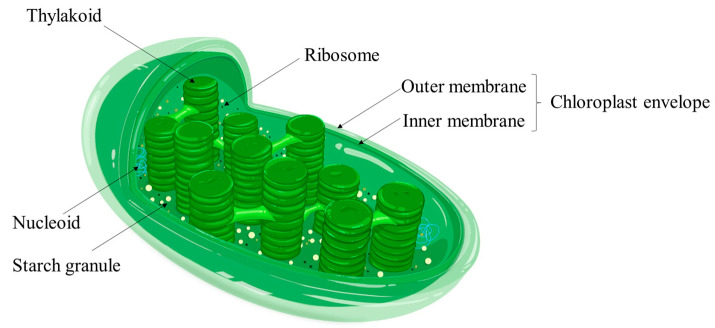
The structure and composition of chloroplasts in plants.

Duckweed can be used as a new type of biomass energy primarily because of its high starch content. Thus, the metabolism pathway of duckweed starch is crucial to further improve energy production. Like other higher plants, duckweed can synthesize starch through photosynthesis [44]. A part of the synthesized starch undergoes material metabolism and energy metabolism, and the rest is stored in the chloroplast in the form of starch granules (Figure 2). Normally, duckweed synthesizes starch under light conditions and decomposes starch stored in chloroplasts for heterotrophic metabolism under dark conditions.

### 3.1. Synthetic Pathway of Duckweed Starch

**Figure 3:**
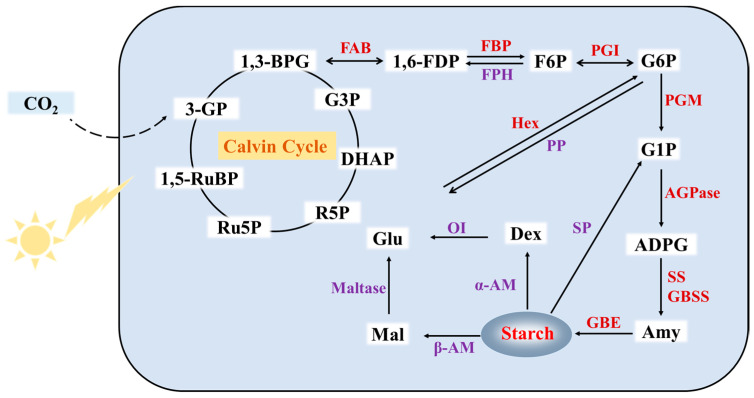
The metabolic pathway of duckweed starch. 3-GP: 3-phosphateglycerate; 1, 3-BPG: 1, 3-bisphosphoglycerate; G3P: glyceraldehyde -3-phosphate; DHAP: dihydroxyacetone phosphate; R5P: ribose-5-phosphate; Ru5P: ribulose-5-phosphate; 1, 5-RuBP: ribulose-1, 5-disphosphat; 1, 6-FDP: fructose-1, 6-diphosphate; F6P: fructose-6-phosphate; G6P: glucose-6-phosphate; G1P: glucose-1-phosphate; ADPG: adenosine diphosphate glucose; Amy: amylose; Mal: maltose; Dex: dextrin; Glu: glucose; AGPase: ADP-glucose pyrophosphorylase; GBSS: granule starch synthase; SS: starch synthase; α-AM: alpha-amylase; β-AM: beta-amylase; SP: starch phosphorylase; PGM: phosphoglucomutase; PGI: phosphoglucose isomerase; FBP: fructose-1, 6-bisphosphatase; FBA: fructose-1, 6-bisphosphate aldose; FPH: fructose phosphate hexokinase; Hex: hexokinase; PP: 6-phosphoglucose phosphatase; OI: isoamylase; GBE: glucan branching enzyme.

Under light conditions, plants absorb CO_2_ and fix CO_2_ through the Calvin cycle for photosynthetic carbon assimilation. Glyceraldehyde-3-phosphate (G3P), the intermediate product of the Calvin cycle, produces fructose-1, 6-diphosphat(1,6-FDP) by the action of the fructose-1, 6-bisphosphate aldose (FBA) enzyme; 1,6-FDP produces fructose-6-phosphate (F6P) by the action of the fructose-1, 6-bisphosphatase (FBP) enzyme; F6P produces glucose-6-phosphate (G6P) by the action of the phosphoglucose isomerase (PGI) enzyme; G6P produces glucose-1-phosphate (G1P) by the action of the phosphoglucomutase (PGM) enzyme; G1P produces adenosine diphosphate glucose (ADPG) by the action of the ADP-glucose pyrophosphorylase (AGPase) enzyme; ADPG produces amylose (Amy) by the action of the starch synthase (SS) and granule starch synthase (GBSS) enzymes; due to the action of the glucan branching enzyme (GBE) enzyme, Amy produces starch (Figure 3).

Studies on the starch synthesis pathway of duckweed showed that the key enzymes required for the synthesis of starch by duckweed differ under different treatment conditions. Huang et al. [33] studied the causes of the increase in starch in duckweed under oligotrophic conditions and found that the activities of key enzymes AGPase, GBSS, and SSS, increased significantly during starch synthesis. Transcriptome analysis showed that the gene expression of these key enzymes was upregulated. Yu et al. [45] found through transcriptome analysis that duckweed-encoding AGPase and SSS transcripts were significantly upregulated under nitrogen starvation conditions. The transcript encoding GBSS was not significantly different under nitrogen starvation conditions. Yang et al. [18] found that under the treatment of different concentrations of pollutants, the starch synthase of duckweed was also affected to different degrees, which ultimately affected the accumulation of starch. Zhu et al. [46] found by RNA-seq that maleic hydrazide treatment might affect the expression of transcripts of some key enzymes in the starch biosynthesis pathway. The expression of these genes was further detected by qRT-PCR, and it was found that AGPase, GBSS, and SS were upregulated by MH treatment.

### 3.2. Degradation Pathway of Duckweed Starch

Under dark conditions, the starch accumulated in chloroplasts is degraded to provide energy to cells. The degradation pathway of starch in plants includes two pathways, i.e., the hydrolysis pathway and the phosphorolysis pathway. The hydrolysis pathway is mainly associated with the α-AM and β-AM enzyme hydrolysis of starch, while the phosphorolysis pathway is mainly associated with the SP enzyme phosphorylation of starch. Through these two pathways, a part of the available starch is transferred to molecules, including Ma, Dex, and G1P, that are further metabolized to Glu and finally undergo anaerobic decomposition to provide energy to the cells.

There are also many reports on the degradation pathway of starch in duckweed, and different treatment conditions have different effects on the enzymes related to duckweed starch degradation. Liu et al. [47] found that the activity of the α-AM enzyme changed slightly relative to the activity of the enzyme in the control group under Uniconazole (plant growth regulator) treatment, and the expression of the β-AM enzyme increased. These results contradicted the increase in starch accumulation under Uniconazole treatment, and physiological data suggested that it was due to the activity of the β-AM enzymes, which was too low to be comparable to that of starch biosynthesis. Yu et al. [45] found a correlation between the downregulation of the expression of duckweed SP and α-AM enzymes and the increase in starch content during nitrogen deficiency.

## 4. Pathways to Promote the Accumulation of Duckweed Starch

Starch is one of the main storage compounds of duckweed and usually accumulates under stress (such as nitrogen deficiency). This is an adaptation in duckweed acquired through the evolutionary process which enabled it to survive in harsh environments [32]. In this study, we reviewed the reported ways to increase the starch content of duckweed, which varies from 2.14% to 78% (DW) according to the species and growth conditions of duckweed. In previous studies, only the effects of nutrient deficiency, application of ABA, and addition of cations on the promotion of starch accumulation have been studied. We further systematically reviewed the effects of different methods. Starch accumulation was promoted mainly by adjusting nutritional conditions and adding exogenous hormones (low cost and easy operation). The culture was conducted under suitable light, and CO_2_ was added to achieve higher starch accumulation (high cost).

### 4.1. Nutritional Regulation Promotes Duckweed Starch Accumulation

Several studies have shown that nutrient deficiencies (such as the deficiency of nitrogen, phosphorus, etc.) can increase the starch content in duckweed (Table 2). Yu et al. [45] found that under nitrogen starvation conditions, starch accumulation in *Lemna aequinoctialis* increased significantly from the initial level of 20% to about 60% (DW). Guo et al. [34] obtained similar results in *Landoltia punctata*, which could rapidly accumulate starch to 52.37% (DW) under nitrogen deficiency. Reid and Bieleski [48] found in 1970 that the effect of phosphorus deficiency on *La. punctata* is similar to that found in other higher plants. The growth of *La. punctata* slows down quickly, the photosynthesis rate decreases gradually, the root system stretches, the chloroplast accumulates starch, and the starch content increases to around 75% (DW). Li et al. [49] showed in 2021 that under phosphorus starvation conditions the total phosphorus content of *La. punctata* decreased while the total carbon content increased. After 15 days of phosphorus starvation, biomass production increased from 12.64 g m^−2^ to 123.87 g m^−2^, while phosphorus utilization efficiency increased to 761.78 g m^−2^, starch content increased from 2.14% (DW) to 38.05% (DW), and the starch yield reached 47.14 g m^−2^. Zhao et al. [35] also studied the effects of nitrogen deficiency and phosphorus deficiency on the starch accumulation in *La. puncata* and found that the starch content of duckweed increased to 23.0% (DW) and 32.5% (DW) under phosphorus deficiency and nitrogen deficiency, respectively, indicating that nitrogen deficiency can promote the starch accumulation in *La. punctata* more than phosphorus deficiency.

Additionally, methods for increasing duckweed starch content through various nutrient deficiencies have also been widely reported. Cheng and Stomp [14] transferred *S. polyrhiza* from a nutrient-rich medium to tap water for five days and found that the starch content of duckweed increased from 20% (DW) to 45.8% (DW). Xu et al. [15] transferred *S. polyrhiza*, which was grown in a diluted pig manure culture pond, to well water on a pilot scale. After 10 days, the starch content of duckweed increased to 64.9% (DW), and the annual output of starch was as high as 9.42 × 10^–3^ kg ha^–1^. Zhao et al. [50] treated *Lemna minor*, *La. punctata, Lemna perpusilla*, and *S. polyrhiza* with 5% pig farm sewage, and the highest starch content reached 32.9%, 44.4% (DW), 38.0% (DW), and 35.5% (DW). Ge et al. [51] found that low-nutrient agricultural wastewater could yield *Le. minor* with 36% starch. Tao et al. [52] and Huang et al. [33] transferred *La. punctata* from Hoagland culture medium to distilled water and found that the starch content reached 45.36% (DW), and the total starch weight increased by 42 times. Kruger et al. [36] found that *La. punctata* obtained 30% (DW) starch content through nutrient deficiency and weak light treatment. Rana et al. [53] found that, after *S. polyrhiza* was cultured in Hoagland medium, the starch content increased to 78% (DW), which was the highest starch content of duckweed ever reported.

### 4.2. Application of Exogenous Hormones to Promote Starch Accumulation in Duckweed

Exogenous hormones can increase the starch content of duckweed. Studies have shown that 6-BA, ABA, and uniconazole are the main hormones with strong effects to increase the starch content of duckweed (Table 3).

Jong and Veldstra [54] first found that adding 6-BA was beneficial to the accumulation of starch in *Le. minor*. Smith [55] found that adding ABA to the growth medium of *Le. minor* also increased the starch content. Wang and Messing [56], Wang et al. [57] and Liu et al. [58] found similar patterns in *S. polyrhiza* and *La. punctata*, and the starch content increased to 60% (DW) and 46.18% (DW), respectively, by applying ABA. Liu et al. [47] showed that applying uniconazole promotes the accumulation of starch in *La. punctata*. These results strongly suggested that this phenomenon might be due to the change in the endogenous hormone levels [59]. Chen et al. [60] studied the effects of different concentrations of IAA on the growth of and starch accumulation in *La. punctata*, indicating that low concentrations of IAA can effectively promote chlorophyll biosynthesis and duckweed photosynthesis, which are beneficial to biomass accumulation. Moreover, a high concentration of IAA can increase starch percentage and total starch accumulation by enhancing the activity of AGPase. Liu et al. [61] systematically studied the effects of five kinds of plant hormones, including 2,4-D, 6-BA, ABA, GA3, and BL, on biomass and starch accumulation of *La. Punctata*, and found that 6-BA and ABA were the most effective plant hormones to increase biomass and starch accumulation. These results might provide valuable information for studies on the large-scale application of plant hormones in the production of bioethanol from duckweed.

**Table 3:** The application of exogenous hormones promotes starch accumulation in duckweed.

Phytohormone	Species	Starch Content (DW)	Refs
6-BA	*Le. minor*	69%	[54]
	*La. punctata*	4.59~5.03%	[61]
ABA	*Le. minor*	100 µg g^−1^~600 µg g^−1^ (FW)	[55]
	*S. polyrhiza*	60%	[56]
		125~35.3%	[57]
	*La. punctata*	2.29~46.18%	[58]
		4.78~13.73%	[61]
IAA	*La. punctata*	4.5~7.5%	[60]
Uniconazole	*La. punctata*	3.16~48.01%	[47,59]
2,4-D	*La. punctata*	4.39~6.72%	[61]
GA_3_,	*La. punctata*	4.83~5.24%	[61]
BL	*La. punctata*	4.81~4.89%	[61]

6-BA: N6-Benzyladenine; ABA: abscisic acid; IAA: auxin; 2,4-D: 2, 4-dichlorophenoxyacetic; GA_3_: gibberellin 3; BL: brassinosteroids; DW: dry weight; Refs: references.

### 4.3. CO_2_ Supplementation Promotes Starch Accumulation in Duckweed

Studies on the effect of CO_2_ on the accumulation of starch in duckweed are limited, and the conclusions drawn are quite different. Jacobs [62] showed that although extra CO_2_ can increase the photosynthetic efficiency, it also stimulates the growth of *S. polyrhiza*, which eventually leads to the formation of extra photosynthetic products for respiration instead of starch accumulation. Pankey et al. [63] also found similar results. They studied the effect of CO_2_ supplementation on the synthesis of starch in *S. polyrhiza* by growing *S. polyrhiza* in an inorganic nutrient solution at 30 °C and light intensity of 58 µmol m^−2^ s^−1^. Increasing the CO_2_ level by 5% had no significant effect on the starch content. However, Li et al. [64] treated the V-type water (for agriculture), and inferior V-type water in the environment supplemented with CO_2_ for one day, and found that the starch content in *La. punctata* was 32.7% (DW) and 29.1% (DW), respectively, and reached 44.41% (DW) and 37.50% (DW), respectively, after three days, which was significantly higher than that of *La. punctata* in the normal environment. However, on the 15th day, there was a negligible difference in the starch content between the treatments. Therefore, a high concentration of CO_2_ can promote starch accumulation in *La. punctata*, and the effect is more noticeable within a short duration.

### 4.4. Light Source Regulation Promotes Duckweed Starch Accumulation

The accumulation of starch in duckweed is related to the photoperiod (light time/dark time, expressed as L/D), light intensity, and spectral composition (Table 4). Cui et al. (2011) studied the effect of the photoperiod of *S. polyrhiza* on starch accumulation at different temperatures and found that the starch content increased by 50% when the illumination time was extended from L8/D16 to L16/D8 at 25 °C, and the illumination intensity was 40.5 µmol m^−2^ s^−1^; similar results were obtained at 15 °C and 5 °C. Yin et al. [65] studied the effects of different photoperiods (L12/D12, L16/D8, and L24/D0) and light intensities (20, 50, 80, 110, 200, and 400 µmol m^−2^ s^−1^) on the biomass and starch yield of *Le. aequinoctialis* and found that starch accumulation occurred at 110 µmol m^−2^ s^−1^. Chen et al. [66] used *La. punctata* as the material, adopting four different photoperiods (L12/D12, L16/D8, L20/D4, and L24/D0), and found that the accumulation effect of dry matter and starch in duckweed was the best under full illumination. Liu et al. [67] treated *La. punctata* with L24/D0 and oligonutrition simultaneously and found that the starch content was as high as 60.03% (DW). Xu et al. [68] improved the starch content of *S. polyrhiza* by precisely controlling the spectral composition and nutritional status and recorded energy consumption. The energy consumption decreased by 12.56%, and the production efficiency was the highest when the red light/blue light ratio was 4:1.

### 4.5. Turion Induction Promotes Starch Accumulation in Duckweed

An increase in the accumulation of starch in duckweed through dormant body induction was mainly studied in *Spirodela*. McCombs and Ralph [69] first called the turion of *La. punctata*, the state in which *La. punctata* did not grow but accumulated starch in dark culture. The turion of duckweed develops due to the vegetative propagation of duckweed under adverse environmental conditions. Compared to normal leaves, turion is smaller, with less aerenchyma and a thicker cell wall. The turion has been found in *La. punctata, W. globosa*, *W. arrhiza*, *S. polyrhiza* [70]. During the formation of turion, the growth of duckweed is inhibited, and the utilization of sugar is reduced, which causes more sugar to be stored as starch [71]. The activity of ADPGase in the turion of duckweed might be different from that in the thallus. APL2 and APL3, two subunits of ADPGase, are highly expressed in the early stage of turion formation, while APL1 has a low expression in the whole process. Additionally, the activities of α-amylase and β-amylase are inhibited to different degrees in turion [56], which indicates high starch accumulation in turion.

Appenroth et al. [72] reported the standardized process of culturing *S. polyrhiza*, as well as the method of inducing its turion formation and germination. They found that increasing the photon flux rate of blue or red light increased the yield of turion. Xu et al. [68] also showed that nutritional hunger can promote the production of turion, and the optimized spectral composition can significantly increase the production of turion. Zhao et al. [73] showed that the starch content in the dormant body of duckweed increases with the time taken to form the dormant body, and ABA treatment can further increase the starch content of the dormant body by up to 65% (DW). Wang and Messing [56] used ABA to induce *S. polyrhiza* to form the turion for characterizing the transformation of *S. polyrhiza* from the growth stage to the dormancy stage. The process of differentiation of the thallus, which represents the growth stage, to the turion was studied, and their morphology, superstructure characteristics, and starch content were determined. The results showed that the turion is pigmented and rich in anthocyanins and has sufficient density to become submerged in a liquid medium. TEM observation of the turion had smaller vacuoles, smaller intercellular spaces, and more starch granules surrounded by a thylakoid membrane compared to the thallus. After two weeks of ABA treatment, the turion accumulated more than 60% starch in dry biomass.

The turion with such high starch content is a starch production system in duckweed, and can be used for economical biofuel production. Xu et al. [70] systematically studied the physiological, biochemical, and production characteristics of the turion and showed that it is a high-quality ethanol fermentation substrate, as it has a high starch content (65.63%, DW) and a low lignocellulose content (12.82%, DW). The harvested turion was used for ethanol fermentation for the first time, and the ethanol yield was 0.34 g g^–1^ (turion). The feasibility of bioethanol production was preliminarily evaluated. However, it is important to note that the formation of turion is often accompanied by a slowdown in plant growth and a reduction in yield [61,70,71].

### 4.6. Other Stress Factors That Promote the Accumulation of Starch

Besides the abovementioned common ways, other stress factors, such as heavy metals, high salt, ammonia nitrogen, etc., can also be added to create stress adversity for increasing and accumulating starch in duckweed (Table 5).

Sree et al. [74] found that starch increased in the presence of Co^2+^, and the accumulated amount of starch reached the maximum level after four days of treatment. Starch accumulation increased with the increase in Co^2+^ concentration. When the highest concentration was 5.9 mg L^−1^ Co^2+^, it reached 2.9% of the fresh weight of leaves, equivalent to 40.5% of the dry weight of leaves. Guo et al. [75] found a similar pattern. They showed that low concentrations of Co^2+^ and Ni^2+^ (≤0.5 mg L^−1^) can promote the growth of *La. punctata*. Although the growth rate, net photosynthesis rate, chlorophyll content, and Rubisco activity were significantly inhibited at higher concentrations (5 mg L^−1^), the starch content in *La. punctata* increased sharply to 53.3% (DW). These results were attributed to an increase in the activities of AGPase and soluble starch synthase, and a decrease in α-AM enzyme activity after exposure to excessive Co^2+^ and Ni^2+^. Therefore, *La. punctata* can be regarded as a potential material for bioremediation of water polluted by Co^2+^ and Ni^2+^ and the production of high-quality biomass. Zhao et al. [76] showed that the starch content of duckweed increased with an increase in copper concentration, and the starch content of *La. punctata* and *Le. minor* increased from 2.34% and 3.74% to 13.37% and 8.41%, respectively, under 1 mg L^−1^ Cu^2+^ stress.

The photosynthetic organs of duckweed directly face nutrient suppliers (water), and thus, their responses to salt might be different. Cheng [77] studied the effects of salt stress on the growth and antioxidant response of *S. polyrhiza* and found that NaCl significantly reduced the accumulation of photosynthetic pigments and inhibited the growth of plants, which proved that the adaptive active oxygen scavenging system could protect *S. polyrhiza* from oxidative damage under salt stress. Fu et al. [78] found that the fresh weight, Rubisco, AGPase activities, and starch content of *S. polyrhiza* decreased significantly on the first day after treatment with 5.85 g L^−1^ and 8.77 g L^−1^ NaCl, but gradually recovered in the next few days and accumulated more starch from the third to fifth day. Xiao et al. [41] proposed a process for harvesting high-starch duckweed. By adding NH_4_^+^-N to the culture system, the protein of four duckweed species can be converted to starch. Previous studies have shown that dilution of NO_3_^−^-N and NH_4_^+^-N leads to starch reduction [79]. Tian, Fang [80] showed that excess NO_3_^−^-N and NH_4_^+^-N could increase the starch content of *La. punctata* from 3.97% (DW) to 8% (DW) and 26.02% (DW). This showed that the coordination of carbon metabolism and nitrogen metabolism plays an important role in the detoxification mechanism of duckweed. Yang et al. [18] showed that the starch content of *La. punctata* increased to 29.8% (DW) under the joint stress of Naphtalene (NAP) and MC-LR, which suggested that this phenomenon might be related to the destruction of starch degrading enzymes.

**Table 5:** Stress promotes the accumulation of starch in duckweed.

Stress Factor	Species	Starch Content (DW)	Refs
Cu^2+^	*La* *. punctata* *Le. minor*	2.34~13.37% 3.74~8.41%	[76]
Co^2+^	*Le* *. minor*	Up to 40.5%	[74]
Co^2+^+Ni^2+^	*La. punctata*	Up to 53.3%	[75]
NaCl	*S. polyrhiza*	12%	[78]
NH_4_^+^-N	*S. polyrhiza,* *Le. aequinoctialis* *La. punctata*	Up to 32%	[41,80]
NO_3_^−^-N	*La* *. punctata*	4~8%	[80]
MC-LR	*La* *. punctata*	Up to 29.8%	[18]
NAP	*La* *. punctata*	Up to 29.8%	[18]

MC-LR: microcystin-LR; NAP: naphthalene; DW: dry weight; Refs: references.

## 5. Study on the Biomass Transformation of High-Starch Duckweed

Duckweed is also a high-quality raw material for developing bioenergy, such as ethanol, butanol, and biogas, because of its ultra-high biomass accumulation rate, short reproduction cycle, growth in floating water, and ease of harvesting. It is a promising raw material for bioenergy production [81,82].

When duckweed is used as biomass raw material for fermentation, ethanol is the most productive energy material. Cheng and Stomp [14] used amylase to hydrolyze duckweed biomass, and the content of hydrolysate (reducing sugar) was 50.9% of the original dried duckweed biomass. When yeast was used to ferment reducing sugar, the ethanol yield of the original dry duckweed biomass was 25.8%. Ma et al. [83] selected 20 geographically isolated *Le. aequinoctialis*, and *S. polyrhiza* duckweed strains to evaluate their bioethanol production potential. The most productive strain was *Le. aequinoctialis* 6000, with a biomass yield of 15.38 × 1.47 g m^–2^, a starch content of 28.68 × 1.10%, and a starch yield of 4.39 × 0.25 g m^–2^. Patel and Bhatt [84] showed that 1 t of *S. polyrhiza* biomass could produce 79.7~80.4 kg starch, and 38.8~40.8 L ethanol could be produced by further fermentation of the starch. Xu et al. [15] hydrolyzed and fermented high-starch duckweed biomass in a 14 L fermentor, and the starch conversion reached 94.7%, which was about 50% higher than the ethanol production from corn. This suggested that duckweed is a competitive starch source in fuel ethanol production. In a study by Chen et al. [16], to improve the ethanol yield of *La. punctata*, pectinase was used for pretreatment, and the pretreatment conditions (enzyme loading, temperature, and pretreatment time) of duckweed were optimized by response design. The maximum glucose yield reached 218.64 mg g^−1^. Further fermentation experiments showed that the ethanol concentration was 30.8 ± 0.8 g L^−1^, the fermentation efficiency was 90.04%, and productivity was 2.20 g L^−1^ h^−1^. After sewage treatment, starch content in *Le. aequinoctialis* increased, and after fermentation treatment, 95% of sugars were fermented to ethanol with a yield of 0.17 g g^−1^ [85]. These results showed that duckweed biomass can produce a large amount of starch, which can be easily converted to ethanol.

The duckweed biomass material can also be used to produce other types of bioenergy. For example, duckweed treated by anaerobic digestion can be used to produce methane, with a yield of 390 mL of CH_4_ g^−1^ [86]. Calicioglu et al. [87] integrated the biological processes of ethanol fermentation, acidogenic digestion, and methanogenic digestion and transformed duckweed after wastewater treatment into bioenergy to the maximum extent. Moreover, in anaerobic fermentation, the combination of duckweed and microorganism fermentation increased methane production by 51.2%, compared to single duckweed fermentation. Li [88] conducted a study on the production of fuel butanol by fermentation of *La. punctata* using *Clostridium acetobutylicum*. Initially, the starch and monosaccharide compositions of *La. punctata* were analyzed, and it was found that *La. punctata* treated by preliminary fermentation and viscosity reduction could be fermented smoothly. Finally, the residual sugar analysis of the fermented mash showed that it had a high proportion of the target product butanol.

Additionally, transforming duckweed biomass into other products is also a current direction of research. Shen et al. [89] used *Actinobacillus succinogenes* GXAS137 to ferment duckweed to produce succinic acid. When batch fermentation was conducted in a 1.3 L stirred bioreactor, about 57.85 g L^−1^ of succinic acid was produced. The yield of succinic acid produced by hydrolysis of *La. punctata* was about 75.46 g L^−1^, and the yield was 82.87% after the enzyme pretreatment, and semi-synchronous saccharification fermentation was performed for 56 h in a 2 L bioreactor. *Le. minor* can be induced to synthesize a large amount of starch by uniconazole treatment. The yield of glucose can reach 93.4% in a short time after treatment at 180 °C, and the yield of the generated glucose converted into ethyl levulinate can reach 55.2% (400.6 g kg^−1^). When [C_3_H_6_SO_3_HPy]HSO_4_ is used as a catalyst, the efficiency of the process can be increased to 81.8% [90]. The dark fermentation of duckweed can also be performed for bio-hydrogen production, with a maximum yield of 169.30 mL g^−1^ (dry weight). Additionally, the fat yield of oil produced by polyculture of waste generated during fermentation with *Chlorella* can be 33 times higher than that produced by using *Chlorella* alone [91]. Lai et al. [92] studied the viscosity-reducing technology in the fermentation of duckweed to produce lactic acid. They used *Lactobacillus casei* CICC 23184 as the fermentation strain to produce lactic acid. The whole fermentation process only used duckweed as the substrate, and the combination of viscosity-reducing enzyme treatment and fed-batch fermentation was used to produce a high concentration of lactic acid, which laid the foundation for the practical development of lactic acid fermentation with duckweed as the raw material.

## 6. Conclusions and Outlook

In short, duckweed, which bears the advantages of high starch content and “no competition with humans for food and land”, is a new type of non-grain starch raw material with high energy efficiency. Several studies have shown that the starch content of duckweed varies widely among varieties and under different conditions. Therefore, it is a feasible way to promote the growth of specific variants of duckweed and improve the photosynthetic efficiency by changing the culture conditions, thereby promoting the synthesis of starch. Moreover, the establishment of an efficient and stable genetic transformation system for duckweed [83] provides technical support for the use of genetic transformation technology to improve the potential of duckweed for biomass applications [93], which might allow duckweed to be used as a new type of biomass energy. There are relatively few studies that apply the combination of emerging technologies to duckweed, and we can combine advantageous duckweed with histological techniques and genetic transformation to obtain duckweed with higher starch accumulation.

## Figures and Tables

**Table 1:** Comparison of duckweed and other biomass energy.

Plants	Advantages or Disadvantages	Species	Refs
duckweed	rapid growth rate	All species	[12]
	high content of starch	*La. punctata*	[32]
		*Le. aequinoctialis*	[33]
		*S. polyrhiza*	[34]
		*Le. minor*	[35]
		*Le. perpusilla*	[35]
		*S. polyrhiza*	[36]
	low environmental requirements	*Le. minor*	[37]
		*Le. japonica*	[37]
		*La. punctata*	[37]
	high adsorption and transfer capacity of nitrogen, phosphorus, organic matter	*Le. minor*	[28]
		*Le. gibba*	[28]
		*La. punctata*	[28]
		*S. polyrhiza*	[28]
	high adsorption capacity of heavy metals	*Le. minor*	[22]
		*Le. gibba*	[22]
		*La. punctata*	[22]
		*S. polyrhiza*	[22]
		*Le. japonica*	[22]
	no competition with people for food and land	All species	[38]
	low cellulose content	*S. polyrhiza*	[15]
corn	Slow growth rate		[12]
	High nutrient condition		[12]
	competition with people for land		[12]

Refs: references.

**Table 2:** Nutrition regulation promotes starch accumulation.

Nutritional Conditions	Species	Starch Content (DW)	Refs
Nitrogen starvation	*Le. aequinoctialis*	20~60%	[33]
	*La. punctata*	16.97~52.37%	[32]
		8.86~32.5%	[49]
Phosphorus starvation	*S. polyrhiza*	75%	[34]
	*La. punctata*	2.14~38.05%	[48]
		8.86~23%	[49]
Nutrient starvation	*Le. minor*	32.9%,	[35]
		10~36%	[50]
	*Le. perpusilla*	38.0%	[35]
	*La. punctata*	3~45.4%	[44,51]
		30%	[52]
	*S. polyrhiza*	44.4%	[35]
	*S. polyrhiza*	64.9%	[15]
		35.5%	[35]
		20~45.8%	[14]
		78%	[36]

DW: dry weight; Refs: references.

**Table 4:** Light source regulation promotes starch accumulation in duckweed.

Condition	Parameter	Species	Starch Content (DW)	Refs
Light intensity (µmol m^−2^ s^−1^)	20, 50, 80, 110, 200 and 400	*Le. aequinoctialis*	3.86~62.24%	[65]
Photoperiod (L/D)	12:12, 16:8 and 24:0	*Le. aequinoctialis*	3.86~62.24%	[65]
	12:12, 16:8, 20:4 and 24:0	*La. punctata*	7~19.75%	[66]
	8:16, 12:12 and 16:8	*S. polyrhiza*	15~67.5%	[43]
	24:0 and 16:8	*La. punctata*	15.7~60.03%	[67]
Spectrum (R/B)	1/0,0/1,1/2,1/1,2/1 and 4/1	*S. polyrhiza*	65~77.01%(turion) 4.82%~23.19%(frond)	[68]

L/D: light time/dark time; DW: dry weight; Refs: references.
